# Short Chain *N*-acyl Homoserine Lactone Production by Soil Isolate *Burkholderia* sp. Strain A9

**DOI:** 10.3390/s131013217

**Published:** 2013-09-30

**Authors:** Jian Woon Chen, Chong-Lek Koh, Choon-Kook Sam, Wai-Fong Yin, Kok-Gan Chan

**Affiliations:** 1 Division of Genetics and Molecular Biology, Institute of Biological Sciences, Faculty of Science, University of Malaya, Kuala Lumpur 50603, Malaysia; E-Mails: cjw246@hotmail.com (J.W.C.); yinwaifong@yahoo.com (W.-F.Y.); 2 Natural Sciences and Science Education AG, National Institute of Education, Nanyang Technological University, 1 Nanyang Walk, Singapore 637616, Singapore; E-Mails: chonglek.koh@nie.edu.sg (C.-L.K.); choonkook.sam@nie.edu.sg (C.-K.S.)

**Keywords:** CepI/CepR homologs, MALDI-TOF mass spectrometry, *N*-hexanoylhomoserine lactone, tropical soil bacteria, triple quadrupole LC/MS

## Abstract

In the bacteria kingdom, quorum sensing (QS) is a cell-to-cell communication that relies on the production of and response to specific signaling molecules. In proteobacteria, *N*-acylhomoserine lactones (AHLs) are the well-studied signaling molecules. The present study aimed to characterize the production of AHL of a bacterial strain A9 isolated from a Malaysian tropical soil. Strain A9 was identified as *Burkholderia* sp. using matrix-assisted laser desorption ionization-time-of-flight mass spectrometry and 16S rDNA nucleotide sequence analysis. AHL production by A9 was detected with two biosensors, namely *Chromobacterium violaceum* CV026 and *Escherichia coli* [pSB401]. Thin layer chromatography results showed *N*–hexanoylhomoserine lactone (C6-HSL) and *N*–octanoylhomoserine lactone (C8-HSL) production. Unequivocal identification of C6-HSL and C8-HSL was achieved by high resolution triple quadrupole liquid chromatography-mass spectrometry analysis. We have demonstrated that *Burkholderia* sp. strain A9 produces AHLs that are known to be produced by other *Burkholderia* spp. with CepI/CepR homologs.

## 1. Introduction

Quorum sensing (QS) refers to bacterial communication via signaling molecules called autoinducers (AIs) [1]. QS enables bacterial cells to respond and to adapt in unison as a population to ever-changing environmental factors such as the availability of nutrients, defense against other microorganisms that may compete for the same nutrients, and avoidance of potentially dangerous toxic compounds [2]. QS controls collective behavior such as biofilm formation, production of virulence, and antibiotic production [3]. Pathogenic bacteria rely heavily on QS systems to control the expression of genes required for virulence in order to escape the immune response for successful infection [4].

In 1992, a new genus *Burkholderia* was proposed by Yabuuchi *et al.* based on the RNA homology group II of the genus *Pseudomonas* [5]. Members of *Burkholderia* are free-living microorganisms found in diverse environments such as soil, water (including sea water), the rhizospheres of plants, humans, various animal species, and the hospital environment [6]. *Burkholderia* strains are promising candidates for biotechnological applications such as biological control, bioremediation, atmospheric nitrogen fixation, and plant growth stimulation [7,8]. More importantly, most of these strains belong to species of the *B. cepacia* complex (Bcc) which have long been recognized as important human pathogens causing serious infections in the lungs of cystic fibrosis patients. Hence, understanding of *Burkholderia* sp. is significant for developing therapeutic agents to treat these antibiotic-resistant bacteria [9]. In this paper we present the QS activity of *Burkholderia* sp. strain A9 isolated from a Malaysian tropical soil. *N*-acylhomoserine lactones (AHLs) produced by strain A9 were confirmed by high resolution triple quadrupole LC/MS analysis.

## 2. Experimental Section

### 2.1. Bacterial Strains and Culture Conditions

All strains (Table 1) were grown on Luria Bertani (LB) (1% w/v tryptone, 1% w/v NaCl, and 0.5% w/v yeast extract).

The LB agar (LBA) was made by addition of 1.5% w/v Bacto-agar. All bacteria were incubated at 28 °C, except *E. coli* strains which were grown at 37 °C. When necessary, growth media were supplemented with tetracycline (20 μg/mL).

### 2.2. Soil Sampling and Isolation of Bacteria

Soil samples were collected in sterile 50 mL polypropylene tubes from Rimba Ilmu (N03°07.803′, E101°39.473′), Malaysia, on August 2010. They were immediately processed. All large particles and plant materials were removed using sterile forceps and spatula. Then, a soil sample (5 g) was mixed with KGm medium (20 mL) [13] which is a basal medium containing NaCl (1.25 g/L), KCl (0.75 g/L), Na_2_SO_4_ (0.25 g/L), KH_2_PO_4_ (7.5 g/L), MgCl_2_ (0.5 g/L), CaCl_2_ (0.25 g/L), NH_4_Cl (0.3 g/L) and 2-(*N*-morpholino) ethanesulfonic acid (MES, 1.0 g/L). After the basal medium was autoclaved and cooled, filter-sterilized (0.22 μm pore size) FeCl_3_, MnCl_2_ , and ZnCl_2_ solutions were added to the basal medium to final concentrations of 5 mg/L, 2.5 mg/L, and 0.6 g/L, respectively. Finally, 3-oxo-C6-HSL (50 mg/L) was added to the KGm medium as the sole carbon source. This mixture was incubated at 28 °C with shaking (220 rpm). After 48 h, an aliquot of the suspension (150 μL) was inoculated into fresh enrichment medium (3 mL). The same procedure was repeated six times. At the seventh enrichment cycle, a diluted suspension was plated onto 10× diluted LB agar to obtain pure colonies. Preliminary QS screening showed that among the colonies screened, bacterial colony labeled as A9 was isolated subjected to further QS analysis [14].

### 2.3. Strain Identification

#### 2.3.1. Sample Preparation for MALDI-TOF MS and Data Analysis

A fresh single bacterial colony was smeared onto a MSP 96 target polished steel BC plate, overlaid with formic acid (1.5 μL, 70%), and air-dried. Subsequently, the sample was overlaid with MALDI matrix (1 μL, 10 mg/mL of α-cyano-4-hydroxycinnamic acid in 50% acetonitrile/2.5% trifluoroacetic acid) and air-dried again. The target plate was then subjected to MALDI-TOF MS analysis [15].

MS measurements were performed on a Microflex MALDI-TOF (Bruker Daltonik GmbH, Leipzig, Germany) bench-top mass spectrometer (equipped with UV laser at wavelength 337 nm) equipped with the Bruker FlexControl software Version 3.3 (Build 108). The spectra were recorded in the linear positive ion mode and analyzed over a mass range of 2 to 20 kDa. The acceleration voltage was 20 kV. Each spot on the target plate was measured by the MBT-autoX.axe autoExecute method which enables the auto manipulation of the laser emission. Every measurement resulted from six series of 40 laser shots at different positions on the spotted product. The bacterial spectra were then analyzed in the Bruker MALDI Biotyper Real Time Classification (RTC) Version 3.1 (Build 65) software. The dendrogram was created by the standard MALDI Biotyper MSP creation method (Bruker Daltonics, Bremen, Germany), where distance values are relative and are always normalized to a maximum value of 1,000. Using the Biotyper software and taking a list of mass signals and their intensities into consideration, dendrograms were generated by similarity scoring of a set of mass spectra. Dendrograms shown had graphical distance values between species constructed from their reference spectra and a correlation function was used for calculating distance values.

The matching of unknown spectra to the main spectra was evaluated based on dedicated score values. For this, peak information for the main spectrum was transformed to a maximum accessible score value. The results were reported as the best match to the Bruker database with the corresponding score value which was calculated on the final score according to which the identification results were evaluated as follows: if the logarithmic value of the final score was between 2.3 and 3, the isolate was identified at the level of species; for values between 2 and 2.3, the identification was secured at the level of genus and probable species identification; for values between 1.7 and 2, the identification at the level of genus was probable; and for values lower than 1.7, no reliable identification was made [16].

#### 2.3.2. Phylogenetic Analysis Using 16S rDNA

The genomic DNA of strain A9 was extracted using the QIAamp^®^ DNA Mini Kit (Qiagen, Germantown, MD, USA) and used as DNA template for PCR. 16S rDNA gene sequence was amplified using the forward primer 27F (5′-AGAGTTTGATCMTGGCTCAG-3′) and the reverse primer 1525R (5′-AAGGAGGTGWTCCARCC-3′) as described previously [17]. The amplicons were then used for bacterial molecular identification. Nucleotide sequences were compared with GenBank databases using the BLASTN program followed by sequence alignment [18,19]. A phylogenetic tree was generated using the Molecular Evolutionary Genetic Analysis (MEGA) version 5.2 with parameter Neighbor-Joining algorithm and bootstrap for 1,000 re-samplings [20,21].

### 2.4. Detection of Soil Isolate AHL Production

The AHL biosynthesis activity of the soil bacterial strain A9 was assayed in cross-streaking with *C. violaceum* CV026 and *E. coli* [pSB401] on LBA [22]. Any AHL molecules produced by A9 diffused through the agar and induced purple pigmentation in *C. violaceum* CV026 [11]. Bioluminescence produced by *E. coli* [pSB401] was measured [10] using the electron multiplier CCD (EM-CCD) camera (C9100-14; Hamamatsu Photonics K. K., Hamamatsu, Japan), which was placed in a completely dark box. *E. carotovora* GS101 and PNP22 served as positive and negative controls, respectively [12].

### 2.5. AHL Extraction

A9 was grown overnight in LB medium (100 mL) buffered with 50 mM 3-[N–morpholino] propanesulfonic acid (MOPS) to pH 5.5 to prevent spontaneous degradation of AHLs [23]. Cell-free culture supernatant was extracted twice with equal volume of acidified ethyl acetate (0.1% v/v glacial acetic acid). Extracts were concentrated to dryness under vacuum and resuspended in a minimal amount of acetonitrile. AHL extracts were analyzed by measurement of bioluminescence, thin layer chromatography (TLC), and triple quadrupole LC/MS.

### 2.6. Synthetic AHLs

All AHLs were purchased from Cayman Chemical (Ann Arbor, MI, USA). The following AHL standards and derivatives were used: *N*-butanoyl-L-homoserine lactone (C4-HSL), *N*-hexanoyl-L-homoserine lactone (C6-HSL) *N*-octanoyl-L-homoserine lactone (C8-HSL), *N*-decanoyl-L-homoserine lactone (C10-HSL), *N*-dodecanoyl-L-homoserine lactone (C12-HSL), *N*-tetradecanoyl-L-homoserine lactone (C14-HSLs), *N*-(3-oxohexanoyl)-L-homoserine lactone (3-oxo-C6-HSL), *N*-(3-oxooctanoyl)-L-homoserine lactone (3-oxo-C8-HSL), *N*-(3-oxodecanoyl)-L-homoserine lactone (3-oxo-C10-HSL), and *N*-(3-oxododecanoyl)-L-homoserine lactone (3-oxo-C12-HSL). Stock solutions for standards (1 g/L) were prepared in acetonitrile and stored at −20 °C.

### 2.7. Measurement of Bioluminescence

A luminometer-spectrophotometer (Infinite M200, Tecan, Männedorf, Switzerland) was used to measure bioluminescence of cell density. An overnight culture of biosensor *E. coli* [pSB401] was grown in LB supplemented with tetracycline (20 μg/mL). Next, the diluted biosensor (200 μL, 1:100) in LB and AHL extract (1 μL) were added to a microtitre well of a 96-well optical bottom microtitre plate [13,19]. Synthetic 3-oxo-C6-HSL (250 pg/μL) was used as the standard. Acetonitrile was used as negative control. Bioluminescence and optical density were read at 495 nm every 30 min for 24 h [24]. Data were presented as Relative Light Units (RLU)/OD_495_ nm against time, indicating approximate light output per cell.

### 2.8. Thin Layer Chromatography (TLC)

AHL extract and known amounts of synthetic AHLs were applied on a reverse phrase C18 TLC plate (TLC aluminium sheets 20 cm × 20 cm, Merck, Darmstadt, Germany). Synthetic AHLs (C6-AHL, 0.1 μg/μL, and C8-AHL, 5 μg/μL) were used as standards. Chromatograms were developed with methanol:water (60:40, v/v) and then air-dried in a fume hood [11]. The TLC plate was then overlaid with a thin film of LBA seeded with *C. violaceum* CV026 and incubated overnight at 28 °C. The presence of AHL was detected as purple spots on LBA and the results were digitally recorded.

### 2.9. Triple Quadrupole Liquid Chromatography Mass Spectrometry (LC/MS) Analysis

To analyse the extracted AHLs from the spent supernatant of strain A9 and the corresponding synthetic AHLs standards, we use LC-MS/MS method. LC was carried on an Agilent 1290 Infinity LC system (Agilent Technologies Inc., Santa Clara, CA, USA) coupled with an Agilent ZORBAX Rapid Resolution High Definition SB-C18 Threaded Column (2.1 mm × 50 mm, 1.8 μm particle size). The flow rate was 0.5 mL/min at 37 °C and the injection volume was 2 μL. Mobile phases A and B refer to 0.1% v/v formic acid in water and 0.1% v/v formic acid in acetonitrile, respectively. The gradient profile used was as follows (time: mobile phase A: mobile phase B): 0 min: 80:20, 7 min: 50:50, 12 min: 20:80, and 14 min: 80:20. MS detection from UHPLC separated compounds was performed on the Agilent 6490 Triple Quadrupole LC/MS system. Precursor ion-scanning experiments were performed in positive ion mode with Q3 set to monitor for *m*/*z* 102 and Q1 set to scan a mass range of *m*/*z* 150 to *m*/*z* 400. Molecular mass of *m*/*z* 102 refers to the lactone ring thus indicating the presence of AHLs. The LC/MS parameters were as follows: probe capillary voltage set at 3 kV, sheath gas at 11 mL/h, nebulizer pressure 20 psi, desolvation temperature at 200 °C. The Agilent MassHunter software was used for the MS data analysis. Analysis was based on retention index and the comparison of EI mass spectra with standards.

### 2.10. Nucleotide Sequence Accession Number

The 16S rDNA nucleotides sequences of strain A9 was assigned GenBank accession no. KF251109. All other rDNA sequences were obtained from GenBank.

## 3. Results and Discussion

### 3.1. Isolation and Identification of Soil Bacterium A9

**Figure 1.**
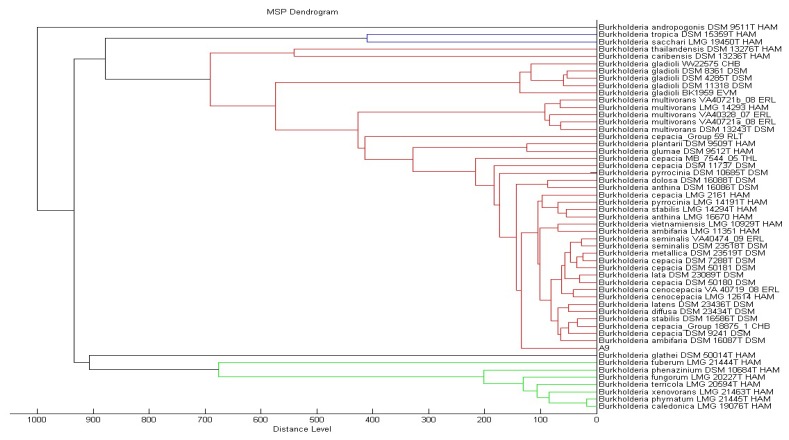
Phylogenetic dendrogram of strain A9 constructed by the standard MALDI Biotyper MSP creation method. A9 is located under *Burkholderia cepacia* group (Bcc).

**Figure 2.**
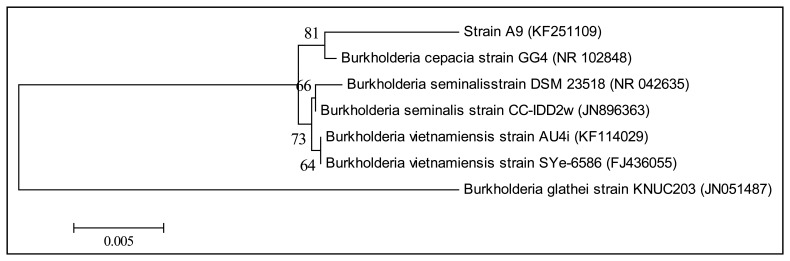
16S rDNA-based phylogenetic tree showing the phylogenetic position of strain A9 with its closest relatives. A total of 1,416 unambiguously aligned 16S rDNA nucleotides were analyzed using Mega 5.2. The horizontal bar at the bottom represents evolutionary distance as 0.005 changes per nucleotide position, determined by measuring the lengths of the horizontal lines connecting the species. *Burkholderia glathei* strain KNUC203 was used as the outgroup. Numbers in parentheses are GenBank accession numbers.

KGm medium supplemented with 3-oxo-C6-HSL as sole carbon source was used to isolate soil bacteria in this study. At each interval of 48 h enrichment cycle, an aliquot of KGm was streaked on diluted LBA to observe bacterial growth. The enrichment cycle was stopped at the seventh cycle. Pure cultures were obtained after several successive streaks from single colonies. Strain A9 was isolated from one of the single colonies and identified using MALDI-TOF MS (Figure 1) and 16S rDNA (Figure 2). Results from MALDI-TOF MS showed that strain A9 belonged to *Burkholderia cepacia* group with 2.18 score value (Figure 1), indicating probably genus identification only [16]. The genus *Burkholderia* currently comprises about 34 species, at least nine of them belonging to *B. cepacia* complex (Bcc). Bcc is a collection of genetically distinct but phenotypically similar *Burkholderia* bacteria [9]. Web-based search and phylogenetic analysis of the 16S rDNA nucleotide sequence of strain A9 showed that A9 possessed 81% similarity to *B. cepacia* strain GG4 (Figure 2) [25].

### 3.2. Detection of AHLs

**Figure 3.**
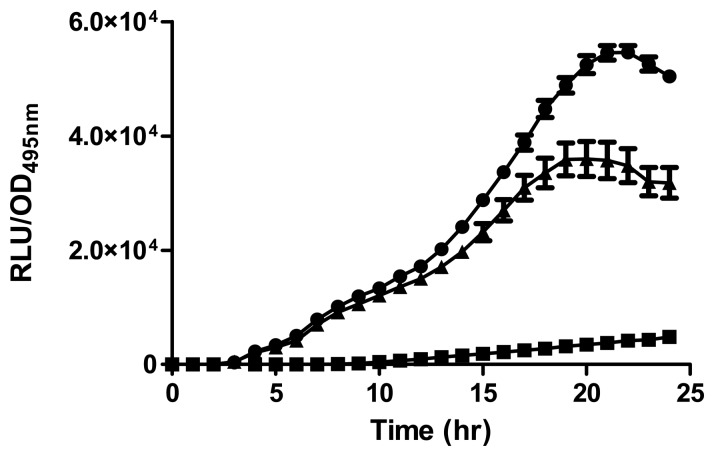
Detection of short chain AHL or AHLs produced by *Burkholderia* sp. strain A9 via bioluminescence of the biosensor *E. coli* [pSB401]. Synthetic 3-oxo-C6-HSL (circle) (positive control), acetonitrile (square) (negative control), and AHL extract from the spent culture supernatant of A9 (triangle) were presented to *E. coli* [pSB401]. Error bars represent the standard error of the mean for three replicates.

**Figure 4.**
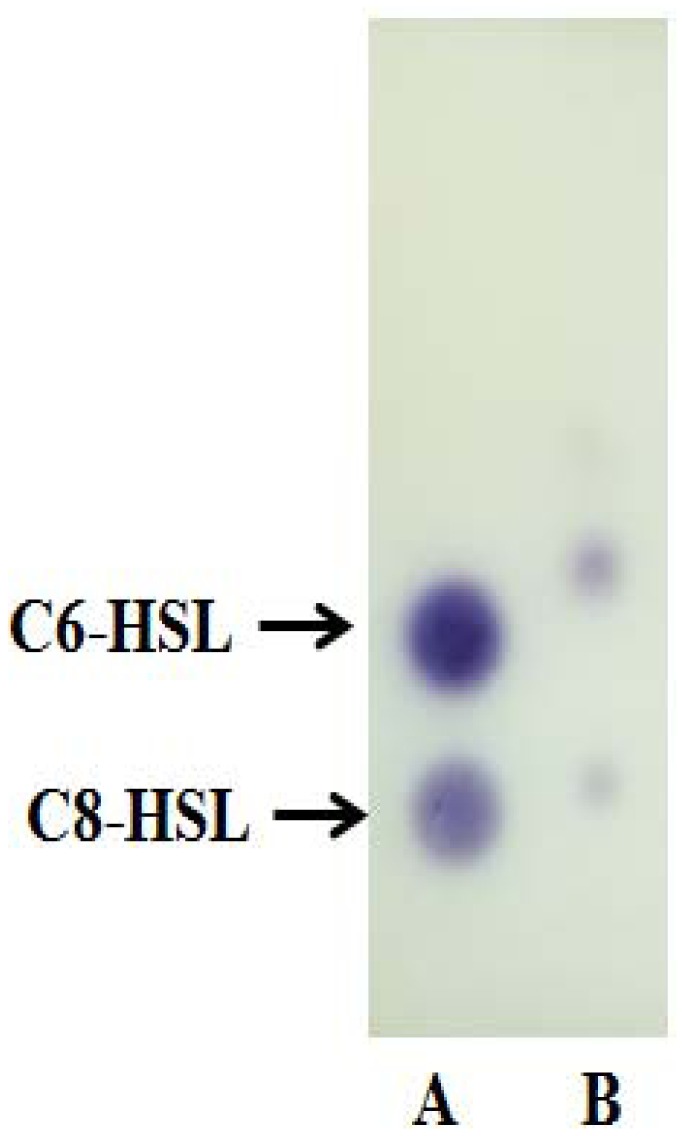
TLC analysis on C18-reverse phase TLC plates chromatographed in methanol/water (60:40, v/v) and visualized with *C. violaceum* CV026. Arrows indicate the positions of AHL standards run on the same plate. Lane A: C6-HSL (0.1 μg/μL) and C8-HSL (5 μg/μL). Lane B: Extract of the spent culture supernatant of A9.

Strain A9 triggered CV026 violacein production and *E. coli* [pSB401] bioluminescence (data not shown) suggesting production of short chain AHLs. The production of AHLs by strain A9 was confirmed by the activation of bioluminescence of *E. coli* [pSB401] (Figure 3). Based in TLC chromatography, the AHL molecules produced by A9 were identified as C6-HSL and C8-HSL (Figure 4). TLC results of the well chromatographed AHL extracts of the spent culture supernatant from strain A9 revealed two well-resolved spots with relative migration factor (R*_f_*) values corresponding to synthetic AHLs (C6-HSL and C8-HSL).

**Figure 5.**
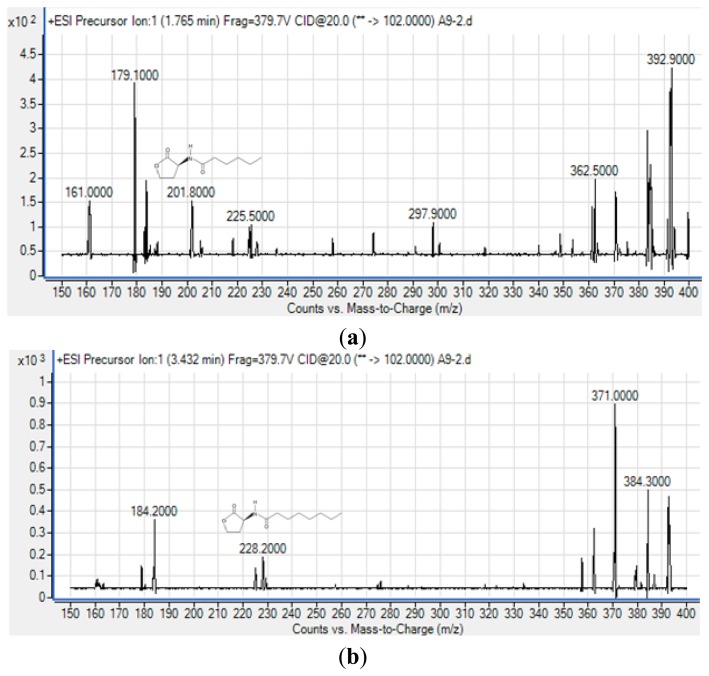
Identification of (**a**) C6-HSL (*m*/*z* 201.8000) and (**b**) C8-HSL (*m*/*z* 228.2000) from AHL extract of the spent culture supernatant of strain A9 by Triple Quadrupole LC/MS.

To obtain more reliable identification of these AHLs, we used high resolution triple quadrupole LC/MS system (Figure 5).

The precursor ion scanning (*m*/*z* 102) on the Agilent 6490 Triple Quadrupole LC/MS system was performed to screening for the lactone ring which is characteristic of the AHL molecules. This method allowed us to identify various AHLs based on detection of the homoserine lactone ring moiety fragmented in the collision cell [26]. Subsequently, the MS results (Figure 5) confirmed the presence of C6-HSL (*m*/*z* 201.800) and C8-HSL (*m*/*z* 228.200) from extract of spent culture supernatant strain A9. The mass spectra for both AHLs are indistinguishable from those of the corresponding synthetic standards that were analyzed in parallel under the same mass spectrometry conditions (supplementary Figures 1 and 2) providing evidence of the production of these AHLs by strain A9. The detection of AHLs, notably C8-HSL, is in agreement with reported work as the AHL QS in the *Burkholderia* Bcc group consists of *luxI*/*R* homologs known as *cepI* and *cepR* that produced predominantly C8-HSL [27,28]. Also, *cepI* synthesizes C6-HSL in addition to C8-HSL which is also in agreement to our work [27,28].

## 4. Conclusions/Outlook

Results from both MALDI-TOF and 16S rDNA nucleotide analysis indicated that the bacterial strain A9, isolated from Malaysian tropical soil, belonged to *Burkholderia* sp. Furthermore, A9 was shown to possess QS activity and produce C6-HSL and C8-HSL, as confirmed by TLC and LC/MS analysis.

## Figures and Tables

**Table 1.** Bacterial strains used in this study.

**Strain**	**Description**	**Source/Reference**
*E. coli* [pSB401]	Short chain AHL biosensor, LuxR receptor cognate AHL = 3-oxo-C6-HSL, Tet^R^.	[10]
*Chromobacterium violaceum* CV026	Double mini-T*n5* mutant derived from ATCC31532, produces violacein pigment only in the presence of *N*–acyl side chains of 4–8 carbons.	[11]
*Erwinia carotovora* GS101	QS positive control for CV026 cross streak test.	[12]
*E. carotovora* PNP22	QS negative control for CV026 cross streak test.	[12]
*Burkholderia* sp. strain A9	Soil isolate.	This work
